# Hypoxia Activates Src and Promotes Endocytosis Which Decreases MMP-2 Activity and Aggravates Renal Interstitial Fibrosis

**DOI:** 10.3390/ijms19020581

**Published:** 2018-02-15

**Authors:** Zhengyuan Cheng, Lei Liu, Zhi Wang, Yingying Cai, Qing Xu, Pingsheng Chen

**Affiliations:** Department of Pathology and Pathophysiology, Medical School, Southeast University, Dingjiaqiao 87, Gulou District, Nanjing 210009, China; 230159257@seu.edu.cn (Z.C.); 230169250@seu.edu.cn (L.L.); 230139667@seu.edu.cn (Z.W.); 220163027@seu.edu.cn (Y.C.); 220163024@seu.edu.cn (Q.X.)

**Keywords:** renal interstitial fibrosis, hypoxia, endocytosis, MMP-2 activity

## Abstract

The aggravation of renal interstitial fibrosis in the advanced-stage of chronic kidney disease is related to decreased matrix metalloproteinase-2 (MMP-2) activity, which is induced by hypoxia in the kidney; however, the specific mechanism remains unclear. We previously demonstrated that inhibition of Caveolin-1, a key gene involved in endocytosis, increased MMP-2 activity in hypoxic HK-2 cells. It has been reported that activated Src (phospho-Src Tyr416) is a key molecule in multiple fibrotic pathways. However, whether Src functions on the regulation of Caveolin-1 and MMP-2 activity in hypoxic HK-2 cells remains poorly understood. To explore the underlying mechanism, a rat model of renal interstitial fibrosis was established, then we observed obvious hypoxia in fibrotic kidney tissue and the protein levels of phospho-Src and Caveolin-1 increased, while MMP-2 activity decreased. Next, we treated HK-2 cells with the phospho-Src inhibitor PP1. Compared with normal cells grown in hypoxia, in cells treated with PP1, the protein levels of phospho-Src and Caveolin-1 decreased, as did the protein levels of the MMP-2-activity-regulated molecules RECK (reversion-inducing-cysteine-rich protein with kazal motifs) and TIMP-2 (tissue inhibitor of metalloproteinase-2), while the protein level of MT1-MMP (membrane type 1-matrix metalloproteinase) increased and MMP-2 activity was enhanced. Therefore, hypoxia promotes the phosphorylation of Src and phospho-Src can enhance the endocytosis of HK-2 cells, which leads to decreased MMP-2 activity and aggravates renal interstitial fibrosis.

## 1. Introduction

Chronic kidney disease (CKD) has become a major global public health burden [1]. Regardless of its cause, CKD ultimately progresses to end-stage renal disease (ESRD), where renal tubular atrophy and interstitial fibrosis are the main pathological changes. Renal interstitial fibrosis is considered to be the final outcome of CKD [2,3]. The etiologies of renal interstitial fibrosis are complicated. Fine et al. proposed the commonly accepted theory of “chronic hypoxia” [4], which proposes that chronic hypoxia is an important factor in the promotion and aggravation of renal interstitial fibrosis. In early-stage CKD, nephron hyperfiltration and a high metabolic rate can cause the peritubular capillary (PTC) network to atrophy and disappear [5,6]. This effect induces hypoxia in the kidney and leads to the development and aggravation of renal interstitial fibrosis. In advanced-stage CKD, intrarenal arteriolosclerosis develops and renal fibrous septa are widely formed; these events induce PTC obstruction and disappearance, blocking the renal interstitial oxygen supply and block oxygen diffusion. These two effects form a vicious cycle, which constantly aggravates interstitial fibrosis [2].

Matrix metalloproteinase-2 (MMP-2) is crucial for the development of renal interstitial fibrosis. In the early-stage of renal interstitial fibrosis, MMP-2 activity increases and the extracellular matrix (ECM) degrades, promoting the mesenchymal transformation of renal tubular epithelial cells, resulting in increased ECM production [7]. Conversely, decreased MMP-2 activity in the advanced-stage of renal interstitial fibrosis results in reduced ECM degradation [7,8]. Ultimately, the imbalance between the synthesis and degradation of the ECM leads to its accumulation in the renal interstitium, resulting in renal interstitial fibrosis. However, the precise mechanism underlying the decrease in MMP-2 activity in the advanced-stage remains unclear. Hypoxia plays an important role in the pathogenesis of renal fibrosis and MMP-2 activation mainly occurs on the surface of the cell membrane [4,8]. Once activated, MMP-2 can exert its effects. Therefore, membrane structure remodeling may affect MMP-2 activity; remodeling is enhanced during hypoxia [9] and endocytosis is an important factor involved in remodeling. Therefore, we hypothesized that the decrease in MMP-2 activity in the advanced-stage of renal interstitial fibrosis is related to the enhanced endocytosis induced by hypoxia. We previously demonstrated that the endocytosis of human proximal renal tubular epithelial cells (HK-2 cells) is enhanced by hypoxia, while MMP-2 activity decreased. However, inhibition of the key gene involved in endocytosis, Caveolin-1, in HK-2 cells increased MMP-2 activity [10,11,12,13]. Accordingly, we suggest that during hypoxia, endocytosis of HK-2 cells is increased, which affects the activation of MMP-2, resulting in decreased MMP-2 activity and aggravation of renal interstitial fibrosis [10,11]. However, the underlying mechanism is not clear.

Src is an important member of the tyrosine kinase family. After using PP1, a highly potent and selective inhibitor of Src [14,15,16,17], inhibition of its activated form phospho-Src (Tyr416) can alleviate renal interstitial fibrosis, making it a new potential target for the treatment of renal interstitial fibrosis. Therefore, we wondered whether a relationship exists between Src, endocytosis and MMP-2 activity during renal interstitial fibrosis. We first established a model of cyclosporine A (CsA)-induced hypoxia in Sprague Dawley (SD) rats. We then examined the protein levels of Caveolin-1, p-Src (Tyr416) and MMP-2 activity, in kidney tissue. Then we used HK-2 cells to explore the underlying relationship between them.

The traditional method of induction CsA-induced renal interstitial fibrosis model in SD rats is CsA treatment accompanied by a low-sodium diet for 28 days [18]. The low-sodium diet (0.05% Na^+^; a normal diet contains approximately 0.4% Na^+^) is crucial for establishment of the model, because it aggravates CsA-induced nephrotoxicity and substantially reduces the time required to develop renal interstitial fibrosis [18,19,20,21]. However, the preparation of a low-sodium diet is time-consuming and expensive. Furosemide is a commonly used drug that is cheap and has a strong sodium-depletion effect and thus can be used to simulate a low-sodium environment. And the diet intake is different in different rats, which will affect the homogenization of the model. However, the gavage method can solve this problem well. Thus, we previously used furosemide instead of a low-sodium diet and a mice model of hypoxic renal interstitial fibrosis was successfully reproduced through intragastric administration in mice by CsA in combination with furosemide [21]. This study is based on our modified approach, using SD rats to establish the renal interstitial fibrosis model.

## 2. Results

### 2.1. Successful Establishment of a Rat Model of Hypoxic Renal Interstitial Fibrosis

#### 2.1.1. Morphology and Renal Function

**Figure 1:**
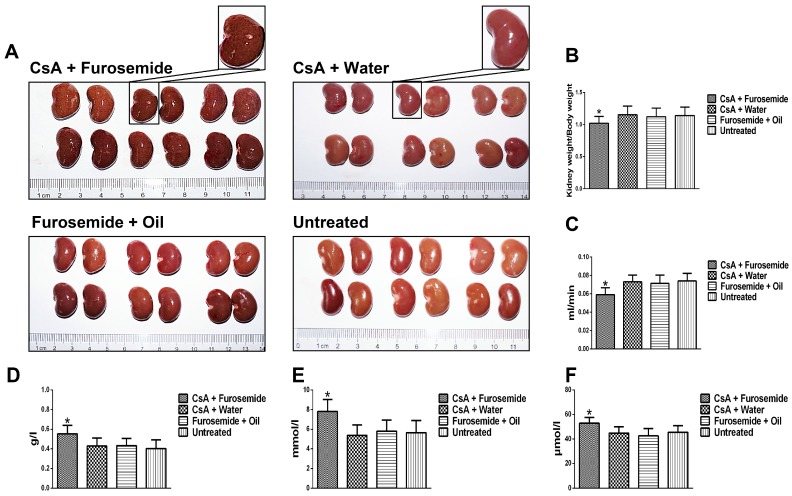
Kidney gross morphology and renal function in different groups on day 14. (**A**) Kidney gross morphology; (**B**) Kidney-to-body-weight ratio (100×). * *p* < 0.05 compared with the other groups; (**C**) Ccr. * *p* < 0.05 compared with the other groups; (**D**–**F**) Urine protein, BUN and Scr, respectively. * *p* < 0.05 compared with the other groups.

**Figure 2:**
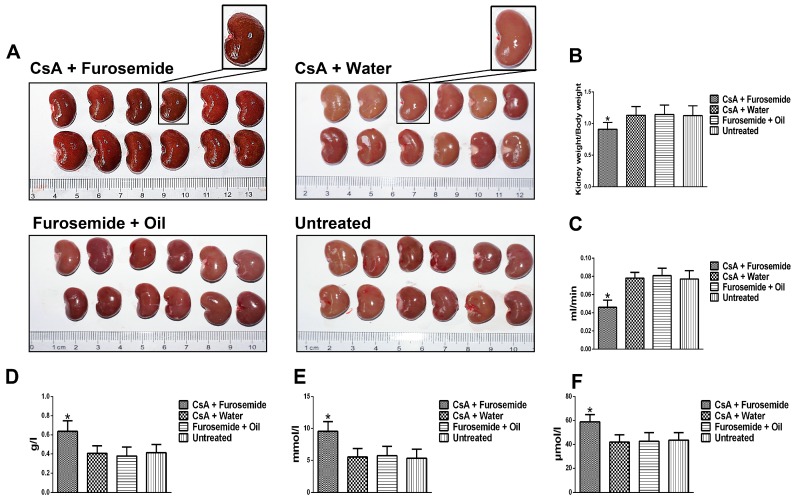
Kidney gross morphology and renal function in different groups on day 28. (**A**) Kidney gross morphology; (**B**) Kidney-to-body-weight ratio (100×). * *p* < 0.05 compared with the other groups; (**C**) Ccr. * *p* < 0.05 compared with the other groups; (**D**–**F**) Urine protein, BUN and Scr, respectively. * *p* < 0.05 compared with the other groups.

On the 14th and 28th days of treatment, kidneys from the rats in CsA + Water, Furosemide + Oil and Untreated groups were the full, ruddy and superficially smooth (Figure 1A and Figure 2A). By contrast, on the 14th day, the kidney tissue from CsA + Furosemide group appeared dark red and was superficially rough and slightly grainy (Figure 1A). On the 28th day, the kidney tissue from CsA + Furosemide group appeared darker and had developed a rough, granular surface (Figure 2A). The ratio of kidney weight to body weight in this group was also markedly decreased compared with the other groups on the 14th and 28th days (Figure 1B and Figure 2B).

On the 14th and 28th days of treatment, no obvious abnormalities were observed in the renal function of rats in CsA + Water, Furosemide + Oil and Untreated groups (Figure 1C–F and Figure 2C–F). However, on the 14th day, the creatinine clearance rate (Ccr) of rats in CsA + Furosemide group was significantly lower than that of rats in CsA + Water, Furosemide + Oil and Untreated groups (Figure 1C), while the urine protein, serum creatinine (Scr) and urea nitrogen (BUN) of rats in CsA + Furosemide group were significantly higher than those of rats in these groups (Figure 1D–F). These profiles on 28th day were more seriously altered than the profiles on 14th day (Figure 2C–F).

#### 2.1.2. Histopathology and Immunostaining

**Figure 3:**
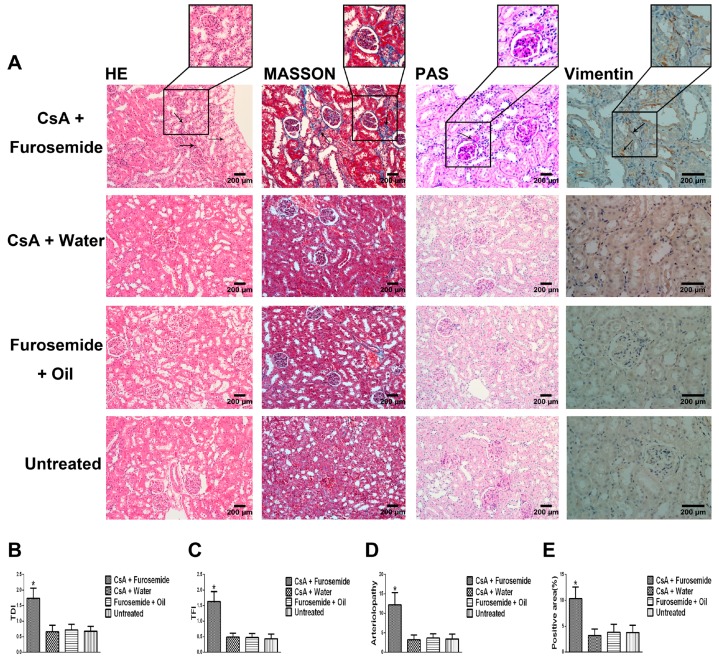
HE, Masson, PAS staining and immunostaining of Vimentin in different groups on day 14. (**A**) HE (200×), Masson (200×), PAS (200×) staining and immunostaining of Vimentin (400×). HE-CsA + Furosemide, arrows denote inflammatory cell infiltration and renal tubular epithelial cell swelling. Masson-CsA + Furosemide, arrows denote collagen deposition. PAS-CsA + Furosemide, arrows denote arteriolopathy. Vimentin-CsA + Furosemide, arrows denote Vimentin positivity. (**B**–**E**) TDI, TFI, arteriolopathy and Vimentin-positive area, respectively. * *p* < 0.05 compared with the other groups.

**Figure 4:**
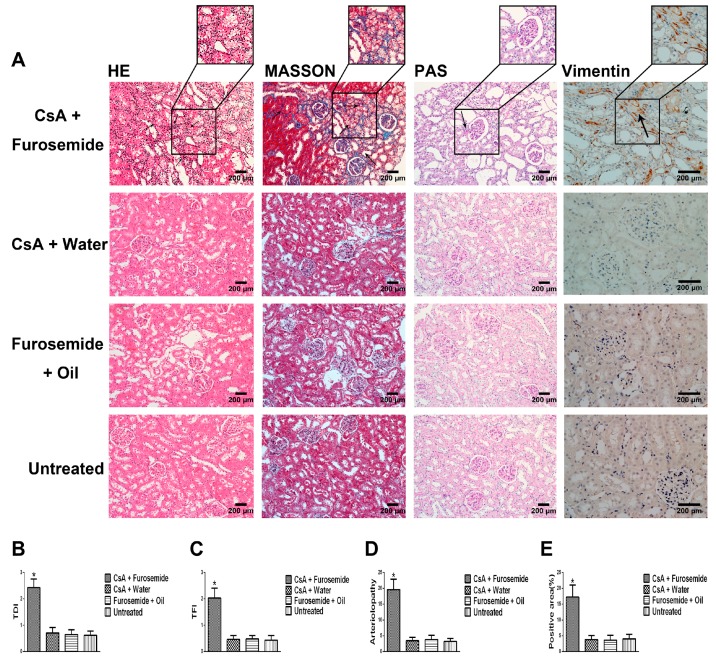
HE, Masson, PAS staining and immunostaining of Vimentin in different groups on day 28. (**A**) HE (200×), Masson (200×), PAS (200×) staining and immunostaining of Vimentin (400×). HE-CsA + Furosemide, arrows denote inflammatory cell infiltration and renal tubular epithelial cell swelling. Masson-CsA + Furosemide, arrows denote collagen deposition. PAS-CsA + Furosemide, arrows denote arteriolopathy. Vimentin-CsA + Furosemide, arrows denote Vimentin positivity. (**B**–**E**) TDI, TFI, arteriolopathy and Vimentin-positive area, respectively. * *p* < 0.05 compared with the other groups.

On the 14th day of treatment, Hematoxylin-eosin (HE) staining of renal tissues from rats in CsA + Furosemide group showed swelling of the tubular epithelial cells, interstitial hyperplasia and inflammatory cell infiltration (Figure 3A); these same parameters were exacerbated on the 28th day (Figure 4A). Masson’s trichrome (Masson) staining showed that clear renal interstitial fibrosis began on the 14th day (Figure 3A) and the fibrosis was more pronounced on the 28th day (Figure 4A). The obvious intrarenal arteriolosclerosis was observed in CsA + Furosemide group on the 14th day by periodic acid-Schiff (PAS) staining (Figure 3A), which progressed further on the 28th day (Figure 4A). However, there were no obvious abnormalities in the renal structures of rats in CsA + Water, Furosemide + Oil and Untreated groups (Figure 3A and Figure 4A). Tubulointerstitial damage index (TDI), tubulointerstitial fibrosis index (TFI) and arteriolopathy were also evaluated (Figure 3B–D and Figure 4B–D).

Immunostaining for Vimentin in rat kidneys from all four groups revealed that the number of Vimentin-positive cells increased notably only in rats in CsA + Furosemide group, which was more distinct on the 28th day than on the 14th day (Figure 3A, Vimentin; Figure 4A, Vimentin). Then the results were evaluated (Figure 3E and Figure 4E).

### 2.2. Protein Levels of Hypoxia Inducible Factor-1α (HIF-1α), Caveolin-1, p-Src (Tyr 416)/Src, MMP-2 and MMP-2-Activity-Regulated Molecules and Detection of MMP-2 Activity in Rat Kidney Tissues

**Figure 5:**
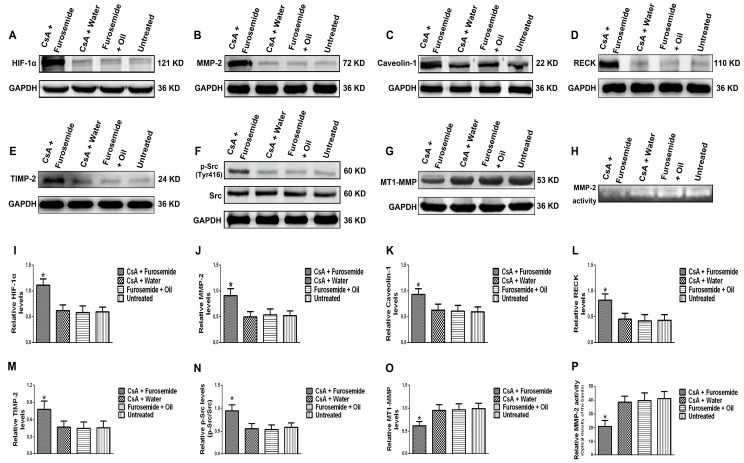
Protein levels of HIF-1α, MMP-2, Caveolin-1, RECK, TIMP-2, p-Src/Src and MT1-MMP and MMP-2 activity in different groups. (**A**–**G**) Protein levels of HIF-1α, MMP-2, Caveolin-1, RECK, TIMP-2, p-Src/Src and MT1-MMP, respectively; (**I**–**O**) Relative protein levels of A-G. * *p* < 0.05 compared with the other groups; (**H**) MMP-2 activity in in different groups; (**P**) Relative MMP-2 activity. * *p* < 0.05 compared with the other groups.

On day 28, the protein levels of HIF-1α, MMP-2, Caveolin-1, p-Src (Tyr 416), tissue inhibitor of metalloproteinase-2 (TIMP-2) and reversion-inducing-cysteine-rich protein with kazal motifs (RECK) in the kidney were higher in rats in CsA + Furosemide group than those in other groups (Figure 5A–F,I–N); however, the protein level of membrane type 1-matrix metalloproteinase (MT1-MMP) decreased in CsA + Furosemide group (Figure 5G,O). No significant changes were observed in the protein level of Src in all four groups (Figure 5F,N). Zymography showed that MMP-2 activity was only decreased in CsA + Furosemide group (Figure 5H,P).

### 2.3. Effect of Src on MMP-2 Activity

#### 2.3.1. Cell Counting Kit-8 (CCK-8) Cytotoxicity Experiment

**Figure 6:**
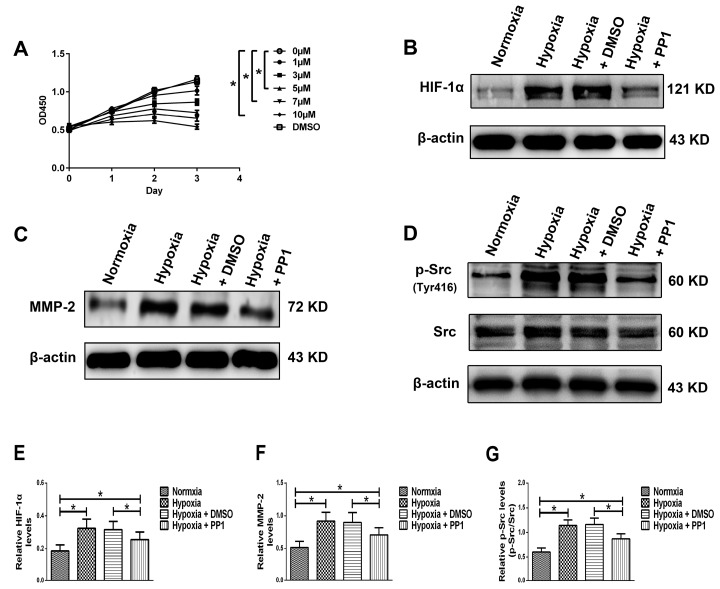
CCK-8 assay and protein levels of HIF-1α, MMP-2 and p-Src/Src in different groups. (**A**) CCK-8 OD450 results. * *p* < 0.05 compared with 0 μM; (**B**–**D**) Protein levels of HIF-1α, MMP-2 and p-Src/Src, respectively; (**E**–**G**) Relative protein levels of (**B**–**D**). * *p* < 0.05 compared with the other groups.

After 24 h, cytotoxicity was seen in HK-2 cells treated with 5, 7 and 10 μmol/L (μM) PP1, a highly potent and selective inhibitor of Src. After 48 h, cytotoxicity was observed in HK-2 cells treated with 3, 5, 7 and 10 μM PP1. After 72 h, cytotoxicity was observed in HK-2 cells treated with 3, 5, 7 and 10 μM PP1 (Figure 6A). However, no toxic effects were seen in HK-2 cells treated with DMSO for up to 72 h (Figure 6A).

#### 2.3.2. Protein Levels of HIF-1α, MMP-2 and p-Src (Tyr 416)/Src in HK-2 Cells

Western blotting showed that in normoxia group and hypoxia + PP1 group, the protein levels of HIF-1α, MMP-2 and p-Src were significantly lower than in hypoxia group and hypoxia + DMSO group (Figure 6B–D,E–G). No obvious change was seen in the protein level of Src among the four groups (Figure 6D,G).

#### 2.3.3. Protein Level of Caveolin-1 and Labelling of Endocytic Vesicles in HK-2 Cells

**Figure 7:**
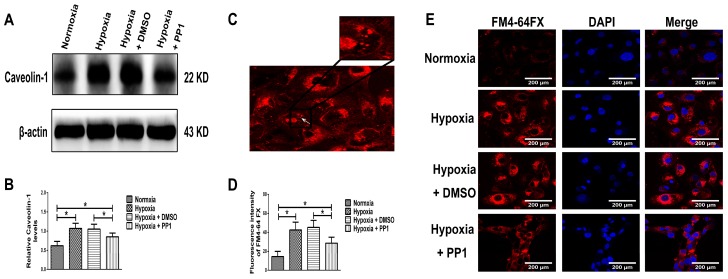
Protein level of Caveolin-1 and observation of endocytosis in different groups. (**A**,**B**) The relative protein level of Caveolin-1 in different groups. * *p* < 0.05 compared with the other groups; (**C**) Endocytic vesicles are shown. The arrow denotes an endocytic vesicle (400×); (**D**) Fluorescence intensity in different groups. * *p* < 0.05 compared with the other groups; (**E**) Endocytosis in different groups (400×).

Western blotting showed that in normoxia group and hypoxia + PP1 group, the protein level of Caveolin-1 were significantly lower than those in hypoxia group and hypoxia + DMSO group (Figure 7A,B). Labelling of endocytic vesicles indicated that compared with cells grown in normoxia, endocytosis was more active in cells grown under hypoxia. After inhibition of Src activation under hypoxia, endocytosis was more active than in cells grown normoxia but still lower than that observed in cells grown in hypoxia without inhibition of Src activation (Figure 7D,E).

#### 2.3.4. Changes in MMP-2-Activity-Regulated Molecules in HK-2 Cells and MMP-2 Activity Detection in Cell Supernatants

**Figure 8:**
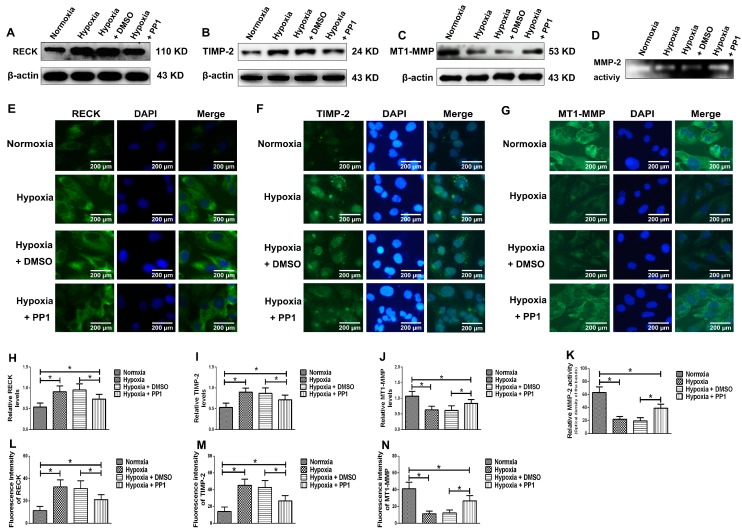
Protein levels of RECK, TIMP-2, MT1-MMP and MMP-2 activity in different groups. (**A**–**C**) Detection of RECK, TIMP-2 and MT1-MMP levels by Western blotting; (**H**–**J**) Relative protein levels of (**A**–**C**). * *p* < 0.05 compared with the other groups; (**D**) The MMP-2 activity; (**K**) Relative MMP-2 activity in different groups. * *p* < 0.05 compared with the other groups; (**E**–**G**) Immunofluorescence detection (400×); (**L**–**N**) Fluorescence intensity of (**E**–**G**). * *p*< 0.05 compared with the other groups.

Western blotting results showed that the protein levels of RECK and TIMP-2 in normoxia group and hypoxia + PP1 group were significantly lower than those in hypoxia group and hypoxia + DMSO group (Figure 8A,B,H,I) but the protein level of MT1-MMP was significantly higher in normoxia group and hypoxia + PP1 group than in hypoxia group and hypoxia + DMSO group (Figure 8C,J). Zymography showed that MMP-2 activity was significantly decreased in cells grown in hypoxia compared with cells in normoxia. However, compared with cells grown in hypoxia only, the MMP-2 activity increased in cells grown in hypoxia and treated with the Src activation inhibitor PP1 (Figure 8D,K). Our immunofluorescence results confirmed the Western blotting findings (Figure 8E–G,L–N).

## 3. Discussion

Since MMP-2 activation occurs mainly on the cell membrane surface [4,8], structural changes in the cell membrane may also influence its activity. Endocytosis is an important mechanism for changing the cell membrane structure and enhanced endocytosis occurs during hypoxia. Caveolin-1 is a key gene involved in endocytosis and up-regulation of Caveolin-1 in HT1080 cells can inhibit MMP-2 activity [12]; however down-regulation of it in cardiac myocytes can enhance the activity of MMP-2 [13]. Our previous study showed that the endocytosis of HK-2 cells is enhanced by hypoxia, which affects MMP-2 activation and results in decreased MMP-2 activity, thus aggravates renal interstitial fibrosis. Here, we studied the role of Src in the changes in MMP-2 activity and Caveolin-1-mediated endocytosis both in vivo and in vitro.

We chose the CsA-induced renal interstitial fibrosis model in SD rats for this study, because this model is not only commonly used but also causes obvious renal arteriolar sclerosis and renal hypoxia [18,19,22]. Therefore, this model is suitable for our research. Our results showed that only the kidneys of rats in CsA + Furosemide group showed obvious fibrosis on days 14 and 28 and the renal function results also suggest that only the renal function of rats in this group was significantly impaired. HE, Masson and PAS staining was performed on renal tissue sections from each group. In CsA + Furosemide group, we observed tubular epithelial swelling, interstitial hyperplasia and inflammatory cell infiltration, obvious renal interstitial fibrosis and intrarenal arteriolosclerosis. However, no notable renal abnormalities were observed in CsA + Water, Furosemide + Oil and Untreated groups. Vimentin is an important marker of fibroblasts and fibroblast proliferation is a typical manifestation of renal interstitial fibrosis [23]. Immunostaining showed substantial fibroblast proliferation in the renal interstitium of rats in CsA + Furosemide group on days 14 and 28 but no obvious proliferation was observed in rats in other three groups. Thus, the traditional pathology, renal function and molecular pathology results indicate that the pathological changes induced in the kidney using our modelling method were similar to those observed using previous methods and similar to those seen in our murine model previously constructed using this method [21]. These findings further demonstrate the feasibility of modelling renal interstitial fibrosis with this method.

The protein levels of HIF-1α, MMP-2, Caveolin-1, p-Src (Tyr416), Src, MT1-MMP, RECK and TIMP-2 and MMP-2 activity were detected in rat kidney tissue. The HIF-1α, a sensitive indicator of hypoxia, was significantly higher in fibrotic renal tissue than in normal tissue. In addition, the protein level of MMP-2 increased in fibrotic renal tissue, as did the protein levels of the MMP-2-activity-regulated molecules, RECK and TIMP-2, while the protein level of MT1-MMP decreased. These results indirectly indicate that although the protein level of MMP-2 increased, its activity decreased. Meanwhile, Zymography results directly indicated that MMP-2 activity decreased. Caveolin-1 is a key gene that mediates endocytosis and increased protein level of Caveolin-1 suggests enhanced endocytosis. Our results showed that the protein level of Caveolin-1 was higher in fibrotic renal tissue than in normal tissue, suggesting that endocytosis was enhanced in fibrotic renal tissue. Phosphorylation of Src can promote renal injury and aggravate renal fibrosis [14,15,16,17] and hypoxia plays an important role in promoting its phosphorylation [24,25]. Src activation was detected in our rat model and we found that the protein level of p-Src (Tyr416) was higher in fibrotic kidneys than in normal kidneys. These data indicate that, compared to the normal renal tissue, Src is activated in the fibrotic renal tissue and the protein level of Caveolin-1 is also increased, while the activity of MMP-2 is decreased. So, does Src play an important role in the Caveolin-1-mediated regulation of MMP-2 activity? Later we performed further study in vitro.

We simulated the chronic hypoxia that occurs in renal interstitial fibrosis by culturing HK-2 cells in hypoxic conditions and used PP1 [14,15,16,17], a highly potent and selective inhibitor of Src, to inhibit Src activation. Based on our previous studies, Yan et al. [14] and CCK-8 results, the PP1 dose and treatment time were set at 5 µM and 24 h respectively. We first examined the protein level of HIF-1α in HK-2 cells. The HIF-1α level was higher in cells cultured in hypoxia than in those cultured in normoxia; studies have shown that increased HIF-1α plays an important role in aggravating renal fibrosis [26]. However, in cells cultured in hypoxia + PP1, compared with cells cultured only in hypoxia, the protein level of HIF-1α decreased; studies have shown that decreased HIF-1α can alleviate renal fibrosis [27]. We also found that the protein levels of MMP-2, Caveolin-1 and p-Src (Tyr416) were higher in cells cultured in hypoxia than in cells cultured in normoxia. However, in cells cultured in hypoxia + PP1, the levels of these proteins were decreased compared with cells cultured in hypoxia only.

MMP-2 activation is closely associated with MT1-MMP, RECK and TIMP-2. MT1-MMP promotes MMP-2 activation and RECK inhibits MMP-2 activation, while TIMP-2 promotes MMP-2 activation when its protein level is low and inhibits MMP-2 activation when its protein level is high [8,28]. Compared with cells cultured in normoxia, the protein levels of RECK and TIMP-2 were higher in cells cultured in hypoxia, while the protein level of MT1-MMP was lower. However, in cells cultured in hypoxia + PP1, compared with those cultured in hypoxia only, the protein levels of RECK and TIMP-2 decreased, while the protein level of MT1-MMP increased. These results were further confirmed by immunofluorescence. These findings indirectly indicate that MMP-2 activity is notably lower in cells cultured in hypoxia than in cells cultured in normoxia. However, in cells cultured in hypoxia + PP1, MMP-2 activity is obviously higher than in cells cultured in only hypoxia. Subsequent Zymography results directly confirmed this finding. We also labelled endocytic vesicles with FM4-64FX dye to directly observe the endocytosis of cells [29] and the results were in accordance with the protein level of Caveolin-1. Studies have shown that endocytosis is enhanced under hypoxia and the enhancement of endocytosis can decrease the protein level of MT1-MMP and cause the protein levels of RECK and TIMP-2 increased [30,31,32]. We obtained same results in this study. In addition, our previous study suggested that under hypoxia and endocytosis inhibition, the protein levels of TIMP-2, MT1-MMP and RECK were the similar as those observed in this experiment. These data show that in hypoxic conditions, Src can affect the protein levels of TIMP-2, MT1-MMP and RECK by affecting endocytosis, thus altering MMP-2 activation.

The pathogenesis of renal interstitial fibrosis is a combination of multiple factors [8]. Upon kidney injury, inflammation leads to inflammatory cell infiltration. In this process, the injured cells and inflammatory cells in kidney release a large number of inflammatory mediators and may further lead to the activation of cell signaling pathways that are related to fibrosis, such as TGFβ1, STAT3 and EGFR [14,15], among others. Eventually, this will lead to ECM deposition in kidney interstitium, which blocks the PTC and induces hypoxia. Src is expressed ubiquitously in the cell and its phosphorylation at Tyr416 can further activate the aforementioned fibrotic signaling pathways, indicating that Src is important in renal interstitial fibrosis. Yan et al. [14] have shown that the renal interstitial fibrosis can be alleviated by intraperitoneal injection of PP1 in mice with renal interstitial fibrosis; the underlying mechanism is that inhibition of Src activation by PP1 can inhibit the fibrotic signaling pathways and reduce ECM production. Meanwhile, our research shows that MMP-2 activity can be increased by blocking endocytosis through inhibition of Src activation by PP1. And increased MMP-2 activity can in turn promote ECM degradation, which also contributes to the reversal of renal interstitial fibrosis [7,8] Therefore, this study further supports the viewpoint that Src is a promising target for the treatment of renal fibrosis. Chemical inhibitors targeting Src have been developed as potential drugs for the treatment of tumors and some of them are already in clinical trials [33]. These drugs may have some therapeutic effects on renal fibrosis but further studies are needed to confirm it.

## 4. Materials and Methods

### 4.1. Study Rats and Reagents

The study was approved by the Animal Care Committee of Southeast University (NO. 20161115007). Eighty male SD rats purchased from Qinglongshan Experimental Animal Company (Nanjing, China) were housed in a facility with controlled air, temperature and light. At the beginning of the experiment, the rats weighed 230–260 g. The rats were provided a normal diet (XieTong, Nanjing, China) and tap water throughout the experiment. CsA (ChemBest, Shanghai, China) was diluted to 10 mg/mL in sunflower oil (Arawana, Shanghai, China). Furosemide (Zhaohui, Shanghai, China) was diluted to 10 mg/mL in distilled water.

### 4.2. Experimental Design

The rats were randomly divided into 4 groups of 20 rats each and received the following interventions: CsA + Furosemide group, received 50 mg/kg furosemide 2 days before the beginning of the experiment, followed by daily administration of 25 mg/kg CsA and 50 mg/kg furosemide on alternate days for a total duration of 28 days. CsA + Water group, received 50 mg/kg distilled water 2 days before the beginning of the experiment, followed by daily administration of 25 mg/kg CsA and 50 mg/kg distilled water on alternate days for a total duration of 28 days. Furosemide + Oil group, received 50 mg/kg furosemide 2 days before the beginning of the experiment, followed by daily administration of 25 mg/kg sunflower oil and 50 mg/kg furosemide on alternate days for a total duration of 28 days. Untreated group, was untreated for all 28 days. The body weight of each rat was recorded every 4 days and the dosage was adjusted accordingly.

After treatment for 14 and 28 days, metabolic cages were used to collect urine and 6 rats from each group were anaesthetized with pentobarbital (Xiya, Linyi, China) and sacrificed. Kidneys were obtained for histology and Western blotting detection; blood was obtained for serum analysis.

### 4.3. Serum and Urine Determinations 

Whole blood was transferred into a separation gel coagulation tube (BD, Franklin Lakes, NJ, USA) and serum was obtained by centrifugation (1400× *g* for 5 min). The Scr and BUN levels were determined using an automatic biochemical analyzer platform (Beckman, Kraemer Boulevard, CA, USA). Urine protein was measured in the same way and the Ccr was calculated.

### 4.4. Histopathology

HE, Masson and PAS stains were used to evaluate the renal pathology of rats in different groups. After euthanasia, the kidneys were immediately removed, photographed and weighed and a portion of each kidney was removed and frozen in liquid nitrogen. The remaining kidney tissue was fixed in 4% paraformaldehyde, embedded in paraffin and sectioned. HE staining was used to highlight changes in kidney architecture and the TDI was determined as described by Shihab et al. [34]. Masson staining was used to study interstitial renal fibrosis and the TFI was evaluated as described by Shihab et al. [34]. Arteriolopathy was evaluated by PAS staining and was calculated as described by Li et al. [35].

### 4.5. Immunohistochemistry 

Interstitial cells were labelled by immunostaining with an anti-Vimentin antibody to reveal fibroblast proliferation in the interstitium. First, the specimens were incubated with the anti-Vimentin primary antibody (1:400; Proteintech, Rosemont, IL, USA) at 4 °C overnight and each slide contained a negative control. Next, the anti-Vimentin primary antibody was visualized using a one-step polymer detection kit (Maixin, Fuzhou, China). Finally, the results were observed and evaluated as described by Liu et al. [36].

### 4.6. Cell Treatment and CCK-8 Cytotoxicity Experiment

HK-2 cells were obtained from the Chinese Academy of Sciences Shanghai Cell Bank (Shanghai, China) and cultured in DMEM (Gibco, Waltham, MA, USA) supplemented with 10% fetal bovine serum (Gibco) in a 37 °C incubator with 5% CO_2_ (Thermo, Waltham, MA, USA). The PP1 dose and treatment time (Selleckchem, Houston, TX, USA) were determined by a Cell Counting Kit-8 (CCK-8, Beyotime, Beijing, China) cytotoxicity experiment. PP1 was dissolved in dimethyl-sulfoxide (DMSO, Beyotime). The cells were divided into 2 groups, a normoxia group and a hypoxia group, with 4 × 10^3^ cells/well in 96-well plates and 200 µL of culture medium in each well. The hypoxia group was divided into 6 groups according to the PP1 dose (0, 1, 3, 5, 7 and 10 μM) and DMSO was used as the control. A hypoxia incubator (Thermo) was used to achieve a hypoxic environment (94% N_2_/5% CO_2_/1% O_2_). After 24, 48 and 72 h, optical density (OD) values were measured at 450 nm by a microplate reader (Biotek, Winooski, VT, USA).

After the treatment dose (5 μM) and time (24 h) were determined, the cells were divided into four groups: normoxia group, normoxia without any treatment; hypoxia group, hypoxia without any treatment; hypoxia + DMSO group, hypoxia with DMSO; and hypoxia + PP1 group, hypoxia with PP1.

### 4.7. Western Blotting 

Fresh rat kidney tissues and HK-2 cells were collected and lysed to extract protein. The protein concentration was measured by a bicinchoninic acid (BCA) protein assay kit (Beyotime). The protein levels of HIF-1α, MMP-2, Caveolin-1, p-Src (Tyr416), Src, MT1-MMP, RECK, TIMP-2, GAPDH and β-actin were measured by Western blotting. Rabbit anti-human antibodies against HIF-1α, Caveolin-1, Src, GAPDH, β-actin (1:1000, Proteintech), MMP-2 (1:1500, Novus, Littleton, CO, USA), p-Src (1:1000, CST, Danvers, MA, USA), MT1-MMP, TIMP-2 (1:1000, Bioworld, St. Louis Park, MN, USA) and RECK (1:1000, CST) were used as primary antibodies and a horseradish peroxidase-linked goat anti-rabbit antibody (1:4000, Proteintech) was used as a secondary antibody. An enhanced chemiluminescence (ECL) imaging method was used to detect the protein bands and the results were analyzed by ImageJ software (Version 1.46, NIH, Bethesda, MD, USA). The experiment was repeated 3 times.

### 4.8. Zymography

Fresh rat kidney tissues were collected and lysed in 0.3% NaCl for protein extraction. The protein concentration was measured by a BCA protein assay kit. Subsequently, the MMP-2 activity in the kidney tissues of each group was detected using an MMP-2 Zymography assay kit (Polygen, Beijing, China). HK-2 cells were cultured in serum-free medium for 24 h, the supernatant was collected and a BCA protein assay kit was used to determine the protein concentration. The MMP-2 activity in the supernatant was also detected by an MMP-2 Zymography assay kit and the results were analyzed by ImageJ software. The experiment was repeated 3 times.

### 4.9. Labelling of Endocytic Vesicles in HK-2 Cells by FM4-64FX

FM4-64FX (Thermo) was diluted to 5 μg/mL in Hank’s balanced salt solution in the dark. HK-2 cells were washed with Hank’s solution and 1 mL of FM4-64FX was added to each dish. Laser confocal microscopy (Olympus, Tokyo, Japan) was used to observe endocytosis. The results were analyzed by Image-Pro-Plus software (Version 6.0, Media Cybernetics, Rockville, MD, USA).

### 4.10. Immunofluorescence

HK-2 cells cultured on coverslips were fixed with 4% polyformaldehyde for 15 min and then washed with PBS. Subsequently, the cells were incubated with primary antibodies against MT1-MMP (1:50, Proteintech), TIMP-2 (1:200, Bioworld) and RECK (1:200, Santa Cruz, Dallas, CA, USA) overnight at 4 °C. The next day, the cells were incubated with an Alexa Fluor 488-conjugated secondary antibody (1:400, Proteintech) for 1 h at 37 °C. 4′,6-Diamidino-2-Phenylindole (DAPI) was used to stain nuclei. Finally, a fluorescence microscope (Olympus) was used to observe the results. The results were analyzed by Image-Pro-Plus software.

### 4.11. Statistical Analysis

Data were expressed as the means ± standard deviation. Comparisons between groups were performed using one-way ANOVA with Bonferroni’s post hoc correction (SPSS 18.0, IBM, Chicago, IL, USA). A value of *p* < 0.05 was considered significant.

## 5. Conclusions

This study provides an alternative approach to establishing rat model of CsA-induced renal interstitial fibrosis. In addition, our results suggest that after inhibition of Src activation, the protein level of Caveolin-1 decreased and endocytosis was reduced, while MMP-2 activity increased. This suggests that Src activation plays an important role in hypoxia, endocytosis and changes in MMP-2 activity. Therefore, this experiment both supports and complements our previous findings: the activation of Src during hypoxia can stimulate the endocytosis of HK-2 cells and influence MMP-2 activation, leading to decreased MMP-2 activity and aggravation of renal interstitial fibrosis. It provides one more way to explain why the MMP-2 activity decrease in the advanced-stage of renal interstitial fibrosis.

## Abbreviations

Src	sarcoma viral oncogene homolog
HIF-1α	hypoxia inducible factor-1α
MT1-MMP	membrane type 1-matrix metalloproteinase
RECK	reversion-inducing-cysteine-rich protein with kazal motifs
TIMP-2	inhibitor of metalloproteinase-2
GAPDH	Glucose 6 Phosphate Dehydrogenase
